# Half-Yearly Abstract of the Medical Sciences

**Published:** 1850-04

**Authors:** 


*Half Yearly Abstract of the Medical Sciences.* By Dr. Ranking, Vol. X. July to December, 1849. Lindsay & Blakiston.

To the enterprising publishers, Messrs. Lindsay & Blakiston, we are indebted for the reprint of this invaluable periodical, containing a digest of the Journals both foreign and domestic. The large amount of useful matter, and the extremely low rate at which it is afforded, cannot fail to recommend it to the profession.

Part 1st devoted to Practical Medicine, Pathology and Therapeutics, is divided into five sections. Section I. General pathology. Section II. Diseases of the nervous system. Section III. Diseases of the respiratory system. Section IV. Diseases of the chylopoietic system. Section V. Diseases of the genito-urinary system. Part 2 is devoted to Surgery, and contains four sections, viz: Section I. Symptomatology and diagnosis of surgical diseases. Section II. Nature and causes of surgical diseases. Section III. Nature and treatment of surgical diseases. Section IV. Rare surgical cases. Part 3 contains an excellent digest on the subject of midwifery and the diseases of women, and on the diseases of children. In addition to these, are Reports on the progress of Medicine, Pathology and Therapeutics, by the editor. A Report on the progress of Surgery, by C. Lockhart Robertson, M. D. A Report on Midwifery and diseases of women and children, by the editor, and a report on Psychological Medicine by the editor. This analysis of the volume will give some idea of the variety and extent of its contents, and to those not having access to the large number of journals at the command of the editor, it may be said, that they have in this carefully arranged periodical the *cream* of them all.

